# Public Interest in Sun Protection and Misinformation: A Google Trends and TikTok Analysis

**DOI:** 10.2196/99635

**Published:** 2026-07-23

**Authors:** Rajyk Bhala, Sriram Ambadi, Reid Oldenburg

**Affiliations:** 1 Department of Dermatology, School of Medicine, University of California San Diego, 8899 University Center Lane, Suite 350, San Diego, CA, 92122, United States, 1 8328077286

**Keywords:** social media, health information, misinformation, TikTok, sunscreen, Google Trends, sun protection, dermatology

## Abstract

In this analysis of Google Trends and TikTok content, public interest in anti-sun protection topics increased in recent years, while non–physician-created content demonstrated similar engagement but lower quality and viewer experience than physician-created content, highlighting a mismatch between public interest and information accuracy.

## Introduction

Social media platforms such as TikTok have become influential sources of dermatologic health information and misinformation [1]. Increasing health misinformation has raised concerns about the reliability of online health information [2]. Google search volume reflects information-seeking behavior, whereas TikTok provides algorithm-driven health information. Together, these platforms provide complementary perspectives on public engagement with sun protection topics and the quality of related information on TikTok.

## Methods

### Overview

We conducted a cross-sectional analysis by choosing the top 30 TikTok videos listed under each of the 3 terms, “sun exposure,” “sunscreen toxic,” and “sunblock,” categorized as neutral, anti-sun protection, and pro-sun protection, respectively. A naïve TikTok account with no previous viewing history was used to minimize the algorithm’s personalization of search results and better reflect content encountered by users. Videos were included if they were relevant to the search term, in English, contained commentary, and were uploaded between May 25, 2021, and January 10, 2026. Google search trends were quantified using the Glimpse extension to obtain worldwide and United States absolute search volume data for these terms from 2020 to 2025 [3,4]. Mean annual search volume was calculated to reduce seasonal variation in search trends, and percent change was determined using 2020 as baseline and 2025 as endpoint [4]. Public interest was operationalized as Google search volume for the chosen terms [4]. Viewer engagement scores were calculated as (likes + comments + shares)/views, and creators were classified as physicians or non-physicians based off creators’ self-classification as an MD or DO in their profile [1]. Physician status was not used as a proxy for video quality or viewer experience. Video quality was determined using DISCERN (1=extensive shortcomings to 5=minimal shortcomings) and viewer experience using the Armstrong Viewer Assessment (AVA; 0=poor to 4=very good) by 2 independent evaluators [1,5]. Both evaluators independently assessed each video using predefined criteria. Discrepancies were resolved through discussion and consensus prior to final analysis. DISCERN assesses the quality and reliability of consumer health information and has been widely applied to social media content [5]. The AVA assesses viewer experience and educational value of dermatological online content [1]. Two-tailed *t* tests compared mean DISCERN and AVA scores between physician and non-physician creators, with statistical significance set at .05.

### Ethical Considerations

This study used publicly available, deidentified data and was exempt from institutional board review.

## Results

**Figure 1.**
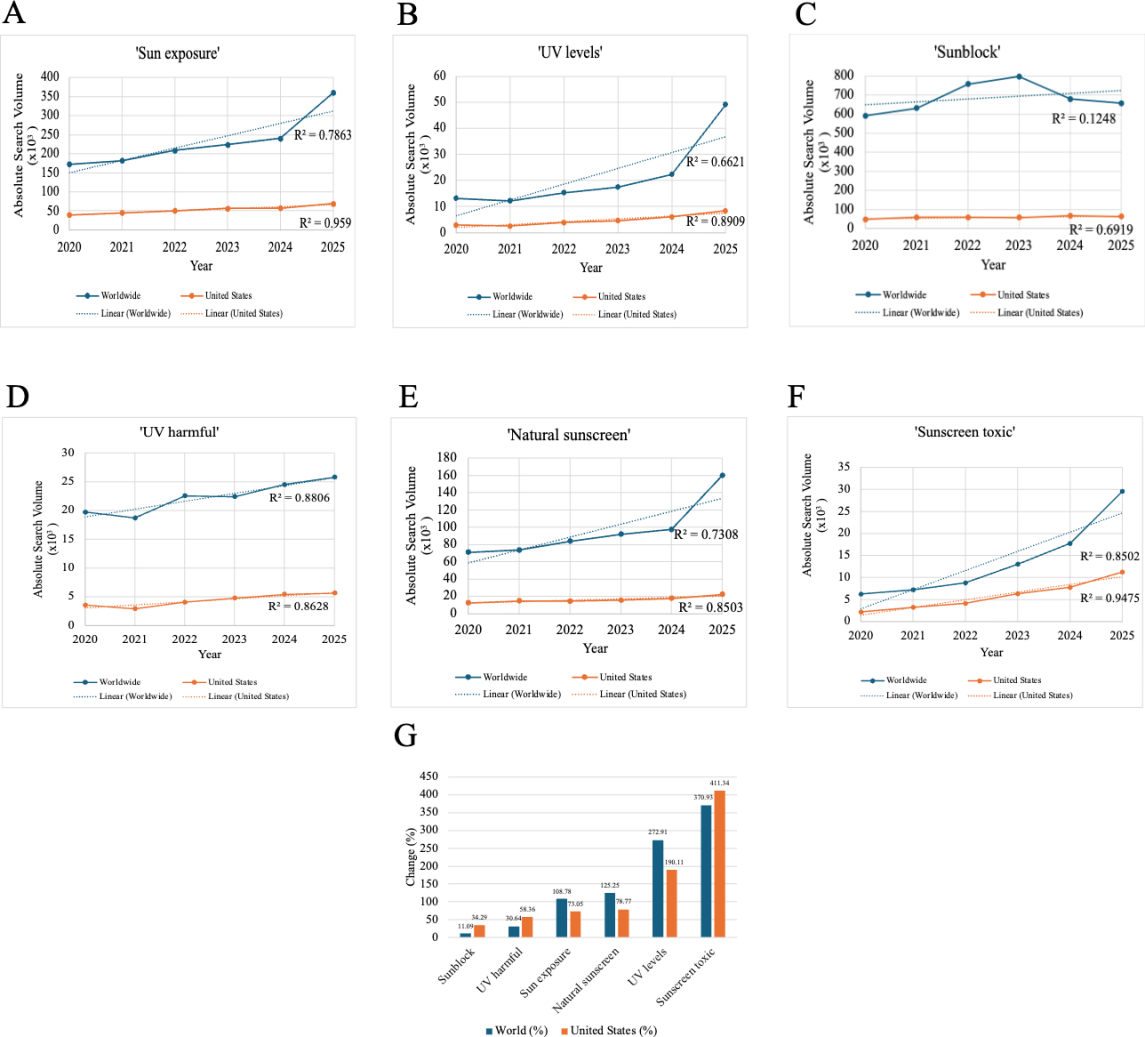
Trends in public interest in sun protection search terms from 2020 to 2025. Panels A–F show annual absolute search volume for the terms “sun exposure,” “UV levels,“ “sunblock,“ “UV harmful,” “natural sunscreen,” and “sunscreen toxic” using Glimpse extension data (accessed February 8, 2025). Panel G summarizes the percent (%) change in absolute search volume between 2020 and 2025. Values are shown for worldwide (blue) and United States (orange) search interest. Absolute search volumes were averaged by year to reduce seasonal variation and obtained using the Glimpse extension.

Google search volume for anti-sun protection terms like “natural sunscreen” (United States: 1.78-fold) and “sunscreen toxic” (United States: 5.1-fold) showed the greatest growth (Figure 1). Neutral search terms like “sun exposure” (United States: 1.73-fold) and “UV levels” (United States: 2.9-fold) revealed moderate growth, whereas pro-sun protection terms reflected the smallest increases by comparison, including “sunblock” (United States: 1.34-fold) and “UV harmful” (United States: 1.6-fold) (Figure 1). Calculated viewer engagement scores did not differ significantly across all 3 terms (sun exposure: 0.12. SD 0.23 vs 0.06. SD 0.02, *P*=.22; sunscreen toxic: 0.06, SD 0.04 vs 0.07. SD 0.04, *P*=.70; sunblock: 0.06, SD 0.04 vs 0.04, SD 0.03, *P*=.10; Table 1). Physician-produced videos achieved higher DISCERN scores across all terms, indicating higher quality (sun exposure: 2.68, SD 2.39 vs 4.10, SD 1.07, *P*<.001; sunscreen toxic: 1.25, SD 0.29 vs 2.75, SD 1.29, *P*=.04; sunblock: 2.68, SD 1.31 vs 4.25, SD 0.38, *P*<.001; Table 1). Physician-produced videos achieved higher AVA scores, indicating better viewer experience (sun exposure: 1.38, SD 1.13 vs 3.10, SD 1.07, *P*<.001; sunscreen toxic: 0.25, SD 0.29 vs 1.75, SD 1.29, *P*=.04; sunblock: 1.68, SD 1.31 vs 3.25, SD 0.38, *P*<.001; Table 1).

**Table 1.** Engagement and quality metrics across sun protection search terms. Viewer engagement, DISCERN quality scores, and Armstrong Viewer Assessment (AVA) scores are reported as mean (SD) for videos associated with the search terms “sun exposure,” “sunscreen toxic,” and “sunblock,” stratified by physician status.

	Videos, n	Engagement, mean (SD)	DISCERN, mean (SD)	AVA, mean (SD)
Sun exposure (n=30)				
Non-physician	20	0.12 (0.23)	2.68 (2.39)	1.38 (1.13)
Physician	10	0.06 (0.02)	4.10 (1.07)	3.10 (1.07)
Sunscreen toxic (n=30)				
Non-physician	24	0.06 (0.04)	1.25 (0.29)	0.25 (0.29)
Physician	6	0.07 (0.04)	2.75 (1.29)	1.75 (1.29)
Sunblock (n=30)				
Non-physician	22	0.06 (0.04)	2.68 (1.31)	1.68 (1.31)
Physician	8	0.04 (0.03)	4.25 (0.38)	3.25 (0.38)

## Discussion

This study uses Google search volume data to measure public interest in sun protection topics [6]. Concurrently, content related to these topics on TikTok may not be accurate or high-quality. Lower DISCERN scores do not necessarily indicate misinformation, but they may reflect incomplete, poorly sourced, or less reliable health information.

These findings reveal a gap between rising public interest in sun protection topics and the quality of readily accessible information. These results are limited by the subjective nature of the DISCERN and AVA methods and the limited sample size. Dermatologists and health care professionals may benefit from addressing misconceptions during clinical encounters, given widespread engagement with sun protection content on social media. Higher-quality, evidence-based content on social media may support informed health decision-making.

## Abbreviations

AVA: Armstrong Viewer Assessment
